# Morphological features of the left atrial appendage in consecutive coronary computed tomography angiography patients with and without atrial fibrillation

**DOI:** 10.1371/journal.pone.0173703

**Published:** 2017-03-13

**Authors:** Miika Korhonen, Johannes Parkkonen, Marja Hedman, Antti Muuronen, Juha Onatsu, Pirjo Mustonen, Ritva Vanninen, Mikko Taina

**Affiliations:** 1 Department of Clinical Radiology, Kuopio University Hospital, Kuopio, Finland; 2 Unit of Radiology, Institute of Clinical Medicine, University of Eastern Finland, Kuopio, Finland; 3 Heart Center, Kuopio University Hospital, Kuopio, Finland; 4 Neuro Center, Kuopio University Hospital, Kuopio, Finland; 5 Department of Cardiology, Keski-Suomi Central Hospital, Jyväskylä, Finland; University of Bologna, ITALY

## Abstract

The majority of intracardiac thrombi form in the left atrial appendage (LAA). Enlargement of this structure, together with certain morphological features, may indicate a predisposition to the formation of thrombi and subsequent cardioembolic stroke. Thus far, studies on LAA morphology have largely focused on those patients with atrial fibrillation (AF). Taking a different approach, we investigated the variation in LAA morphology in a consecutive patient population with and without AF. We evaluated 808 consecutive patients (529 females; mean age 52.5±9.9 years) who underwent coronary artery computed tomography angiography (CCTA), the majority of whom (749) had no history of AF. We assessed the length, lobe number, and morphological classification of their LAAs. Demographic data and medical histories were collated from medical records and then correlated with LAA morphology. The proportions of each of the four morphological classes of LAA for the overall vs. non-AF population were: WindSock, 62.3/61.5%; Cactus, 18.6/18.8%; ChickenWing, 10.0/10.0%; and CauliFlower, 9.2/9.6%. Age (*p*<0.001; *r* = 0.156) and female gender (*p*<0.001) were both found to be associated with an increased body surface area (BSA)-related LAA length. Male patients were more likely to manifest multi-lobed (*p* = 0.003) LAAs, and overweight patients with a greater number of multi-lobed LAA morphological classes (*p* = 0.010). No associations with morphological LAA features could be found for patients with diabetes, hypertension, or dyslipidemia. Nor did the size of the left atrium exhibit any correlation with BSA-related LAA length. In the overall and non-AF populations, aging and female gender were associated with longer BSA-indexed LAAs.

## Introduction

Approximately one in every four cases of ischemic stroke has an underlying cardioembolic mechanism [1]. This fraction may even be an underestimate given that this etiology remains cryptogenic in approximately 25% of all cases [2]. In 90% of cardiogenic strokes, the left atrial appendage (LAA) serves as the site for thrombus formation, prompting extensive medical research into this relatively small structure [3,4].

The most common cause of cardioembolism is atrial fibrillation (AF) [5]. With time, AF alters not only the hemodynamics within the heart, but also simultaneously remodels the left atrium (LA), especially the LAA [6]. Earlier studies have revealed that certain LAA morphologies may be over-represented in patients with cardioembolic stroke [7–9]. The identification of LAA features that might predispose to AF could serve as a useful predictive tool with which to identify patients at an increased risk for paroxysmal AF and, ultimately, life-threatening cardiogenic stroke.

Previous studies on LAA morphology have primarily focused on patients with AF [7–9]. Besides AF, various medical conditions such as coronary artery disease (CAD), valvular diseases, together with age, gender, and obesity, might influence LAA morphology [10–14]. Examining these potential remodeling factors in the absence of AF as a complicating factor would be of considerable value in the design of a predictive screening tool.

The aim of this work was to analyze LAA morphology in patients of different age, gender, and with diverse diagnoses of medical condition. We investigated a large population comprising consecutive patients who had undergone coronary artery computed tomography angiography (CCTA), the majority with no history of AF.

## Materials and methods

All clinical investigations were conducted according to the principles of the Declaration of Helsinki. The Kuopio University Hospital Research Ethics Board approved this study (N:o 82/2004). The Chair of the Hospital District waived the need for written informed consent for these retrospective analyses.

### Study population

The study population comprised consecutive patients admitted to Kuopio University Hospital for CCTA between October 2009 and July 2015. The main indications for imaging were to rule out CAD in patients with a low to moderate pretest probability, to screen for heart failure etiology, or to identify coronary anomalies. This study also included young patients undergoing a preoperative evaluation prior to cardiac valve surgery due to mitral or aortic valve regurgitation, but excluding aortic stenosis. Altogether, 816 patients were imaged. Excluded patients included 3 who were less than 18 years of age, 3 because their LAA could not be reliably assessed from the CCTA image, and 2 patients who suffered AF during CCTA.

### Computed tomography angiography of the coronary arteries

CTA imaging of the coronary arteries was performed in mid-diastole (tube voltage 80–120 mV, 330 mAs) with 64-, 128- slice, and dual energy scanners (Somatom Definition AS 64; Somatom Definition AS+ 128; Definition Edge; Definition Flash, Siemens Medical Solutions, Forchheim, Germany). Collimation was 64x0.6 mm for the Somatom Definition AS 64, and 128x0.6 mm for all other scanners. Following scout acquisition to ensure the precise timing of contrast injection, a test bolus of 10 ml contrast was discharged and measured 5 mm superior to the origin of the left main coronary artery with dual energy scanners; bolus tracking was used for the other scanners. The volume of the contrast agent (Omnipaque 350 mg/ml, GE Healthcare) was 50–80 ml, delivered at an injection rate of 5 ml/s, followed by a 30 mL saline chaser. To achieve a target heart rate of <65 beats per minute, patients with higher initial heart rates were administered 5–20 mg of intravenous metoprolol prior to their examination. Imaging was performed in mid-diastole during sinus rhythm apart from in two patients who suffered from persistent AF. Prospective ECG gating provided helical scan data. Images were reconstructed immediately after scanning; electrocardiographically gated datasets were routinely reconstructed at the 75% phase in the cardiac cycle and, in case of helical scanning, 200–400 ms after the R wave. Our imaging protocol adhered to conventional clinical procedures. The mean effective radiation dose (mSv) during CCTA in the study population was estimated using the conversion factor of 0.028 [15].

### Data assessment

**Fig 1:**
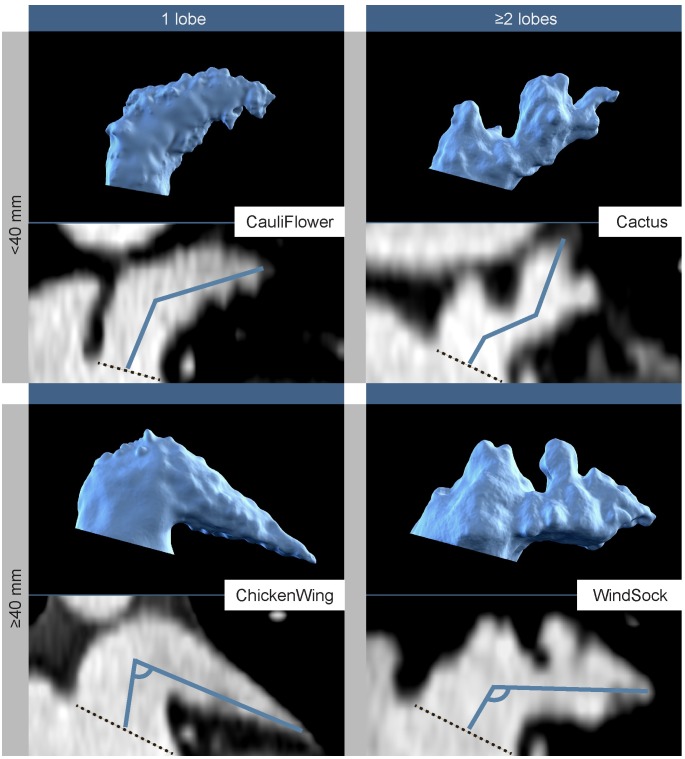
Morphological categories of the Left Atrial Appendage (LAA), as based on Wang’s classification with Kimura’s quantitative qualifiers. The dashed line at the LAA orifice represents the base line from which quantitative measurements were made. The blue line indicates how LAA length and bend angle were derived. Differences between the ChickenWing (<100-degrees) and WindSock category (>100-degrees) are also based on the angle at the proximal part of the LAA.

CCTA images were retrospectively analyzed visually and quantitatively with respect to the length, number of lobes, and morphological classification of the LAA by an experienced observer (MK) using an IDS7 diagnostic workstation (version 17.3.6; Sectra Imtec, Linköping, Sweden). A multiplanar reconstruction provided a three-dimensional perspective. LAA length was measured from the center of the orifice to the most distant point of the LAA, via the center of the main lobe. The LAA bend angle was measured between the axis of the main lobe and the possible secondary lobe (Fig 1). Based on the number of lobes, LAAs were initially classified as one-, two-, or multi-lobed structures. This was followed by a morphological categorization into one of four classes: WindSock, ChickenWing, CauliFlower, or Cactus (Fig 1), as based on the criteria previously described by Wang et al. [16], and Kimura et al. [8].

Demographic information, medical histories, and lifestyle factors were also collated from medical records. The patient was considered to be overweight if this status was either explicitly reported in their medical record, or if their Body Mass Index (BMI), if available, exceeded 25; obesity was defined as a BMI of over 30. Body surface area (BSA) was calculated using Mosteller’s formula [17]. Thereafter, to minimize the influences of body mass and height on LAA length and LA area, relative values were derived by dividing measurements for LAA length and LA area by BSA values.

Diabetes was not classified into subtype. Patients who continuously smoked, or had ceased smoking less than 30 years ago, were considered smokers. The degree of atherosclerosis in coronary arteries was estimated from CCTA analyses performed by experienced radiologists or cardiologists. Non-calcified arteries bore no sign of atherosclerosis whereas stenosis of over 50% was considered significant. LA areas were non-routinely measured from an optimized three-chamber view at the mid-diastolic phase. The severity of valvular regurgitation was classified as either mild, moderate, moderately severe, or severe, based on echocardiography reports. Both moderately severe and severe valvular disease were considered to be of hemodynamic significance and were therefore included in our analyses.

### Statistical analyses

To assess the relationships between age and LAA morphology, the study population was subdivided based on median age, age-related quartiles, or the following age categories: <40 years, 40–49 years, 50–59 years, or ≥60 years.

Continuous variables are presented as mean ±SD, with categorical variables shown as absolute values and percentages. Statistical significance was set at *p*<0.05, with high significance set at *p*<0.001. Spearman’s correlation coefficient was used to investigate the associations between continuous variables, with the Chi-Square test applied to investigate nominal variables. Based on the outcome of the Kolmogorov-Smirnov test, either the Student’s t-test or Mann Whitney U test, were used to compare dichotomized groups for normally distributed or abnormally distributed variables, respectively. The Kruskal-Wallis test was used to analyze continuous variables between multiple groups, with linear regression analyses used to calculate the effects of background factors on relative LAA length and relative LA area as a dependent continuous variable. Data were analyzed using SPSS for Mac (version 22, 1989–2013 SPSS Inc., Chicago, USA).

## Results

### Overall study group

The original study group comprised 808 patients (mean age 52.5 years, 529 women). The majority (n = 749) of these patients had no history of AF. Patients with AF were older, more frequently male, and were more likely to have a history of stroke, TIA, and significant valvular disease. Details of characteristics with comparisons between the AF and non-AF patients are shown in Table 1. The mean effective radiation dose during CCTA was 6.3 mSv.

**Table 1:** Clinical characteristics of the study group.

Characteristic	All patients (N = 808)		Non-AF patients (N = 749)		AF patients (N = 59)		*P* value ^a^
	Value	Analyzed, N	Value	Analyzed, N	Value	Analyzed, N	
Age, years	52.5±9.9	808	52.3±9.8	749	55.0±11.0	59	0.032
Females (%)	529 (65.5)	808	498 (66.5)	748	31 (52.5)	59	0.030
Overweight (BMI>25)	419 (61.4)	682	383 (60.8)	630	36 (69.2)	52	ns
Obese (BMI>30)	163 (31.0)	525	150 (31.3)	479	13 (28.3)	46	ns
Body surface area, m^2^	1.93±0.25	527	1.92±0.25	481	1.99±0.19	46	0.043
Caucasian	807 (100)	808	748 (100)	749	59 (100)	59	ns
Hypertension	409 (50.6)	808	374 (49.9)	749	35 (59.3)	59	ns
Dyslipidemia	488 (64.7)	754	447 (63.9)	700	41 (75.9)	54	ns
Diabetes	42 (5.7)	740	39 (5.7)	690	3 (6.0)	50	ns
Smokers	251 (33.8)	743	231 (33.6)	688	20 (36.4)	55	ns
Sinus rhythm	700 (86.6)	808					
Atrial flutter	5 (0.6)	808					
Paroxysmal AF	58 (7.2)	808					
Chronic AF	1 (0.1)	808					
Non-calcified and non-stenotic coronary arteries	450 (55.7)	808	423 (56.5)	749	27 (45.8)	59	ns
Over 50% stenosis in coronary arteries	142 (17.6)	808	128 (17.1)	749	14 (23.7)	59	ns
Prior myocardial infarction	11 (1.4)	808	11 (1.5)	749	0 (0)	59	ns
Prior stroke or TIA	39 (4.8)	806	31 (4.1)	747	8 (13.6)	59	0.001
Moderately severe or severe mitral regurgitation	6 (0.7)	808	5 (0.7)	749	1 (1.7)	59	ns
Moderately severe or severe aortic regurgitation	16 (2.0)	808	12 (1.6)	749	4 (6.8)	59	0.006
LA area, cm^2^	18.8±5.7	204	18.1±5.1	181	24.2±7.6	23	<0.001
Heart failure	12 (1.5)	808	11 (1.5)	749	1 (1.7)	59	ns

AF, Atrial Fibrillation; BMI, Body Mass Index; LA, Left Atrium; ns, not significant; TIA, Transient Ischemic Attack.

^a^ Statistical significance between AF patients and non-AF patients.

**Fig 2:**
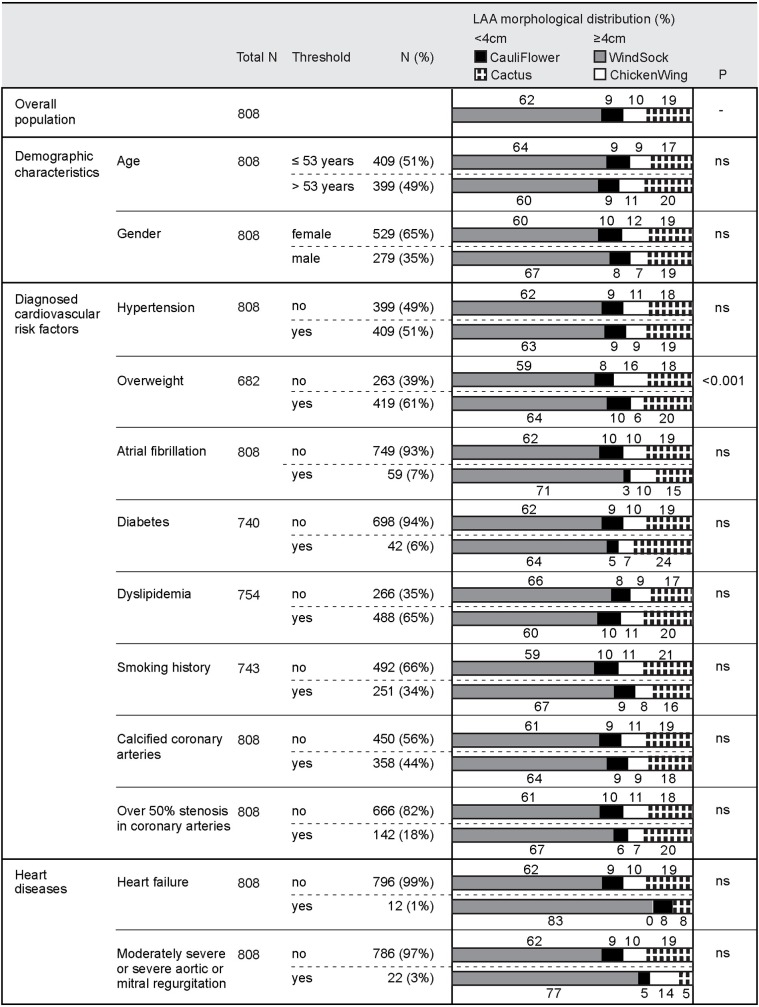
Correlates between morphological classes of the left atrial appendage and classification criteria.

**Fig 3:**
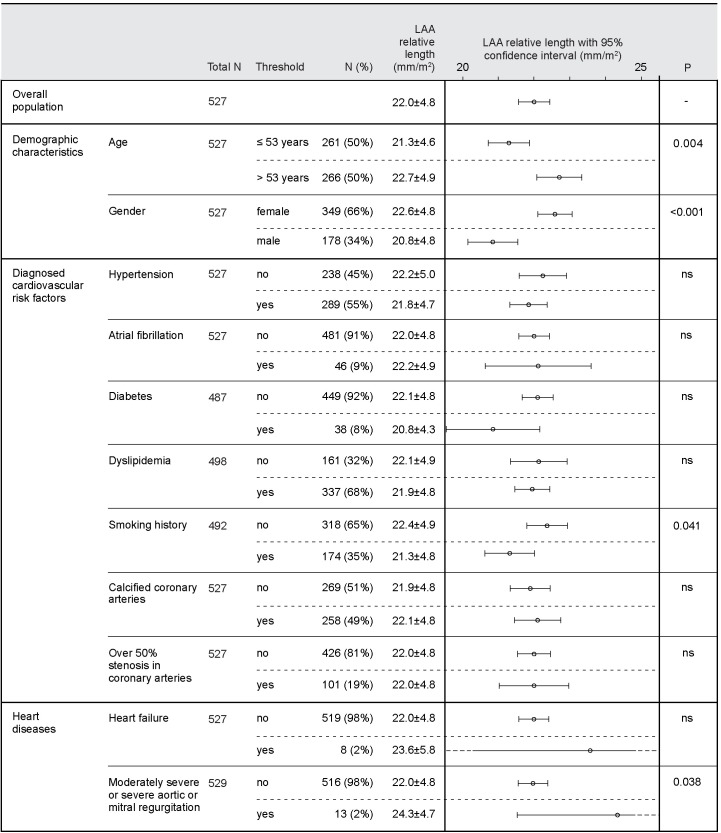
Correlates between relative left atrial appendage length and classification criteria.

**Fig 4:**
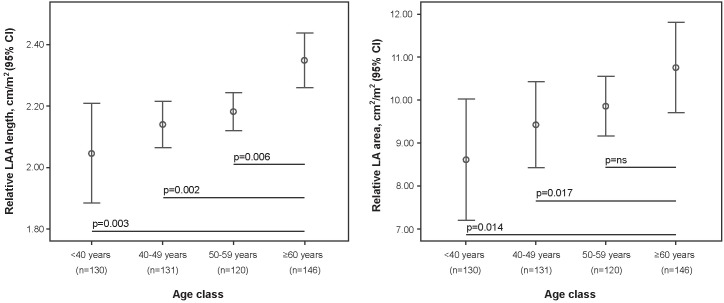
Relative Left Atrial Appendage (LAA) length and relative Left Atrium (LA) area according to age quartile, with 95% confidence intervals.

The prevalence of LAA morphological classes and their relative LAA lengths were analyzed according to the classification criteria detailed in Tables 2 and 3, and illustrated in Figs 2 and 3. Gender (2.26±0.48 cm/m^2^ in female vs. 2.09±0.48 cm/m^2^ in male; *p*<0.001) and smoking (2.13±0.48 cm/m^2^ vs. 2.24±0.49 cm/m^2^; *p* = 0.041) were both significantly associated with relative LAA length, although men were more likely to be smokers than women (46.9% vs. 26.8%; *p*<0.001). As shown in Table 4, a higher age was associated with longer relative LAA lengths (*r* = 0.156; *p*<0.001), and larger relative LA areas (*r* = 0.861; *p*<001). Fig 4 illustrates the differences in relative LAA length and area according to age quartile.

**Table 2:** Associations between the morphological features of the left atrial appendage, the left atrium area, demographic data, and medical histories for the overall study population (n = 808).

Characteristic	N, positives or mean±SD (N, total)	LAA morpho-logical class (*P*)	LAA lobes (*P*)	LAA length (*P*)	Relative LAA length		LA area		Relative LA area	
					N, positives or mean±SD (N, total)	*P*	N, positives or mean±SD (N, total)	*P*	N, positives or mean±SD (N, total	*P*
Females	529 (808)	ns	0.007	<0.001	349 (527)	<0.001	124 (204)	<0.001	74 (124)	ns
Overweight (BMI>25)	419 (682)	<0.001	ns	ns	NA	NA	93 (166)	<0.001	NA	NA
Obese (BMI>30)	163 (525)	ns	ns	ns	NA	NA	38 (122)	0.004	NA	NA
Body surface area, m^2^	1.93±0.25 (527)	0.004	ns	<0.001	NA	NA	1.96±0.25 (124)	<0.001	NA	NA
Hypertension	409 (808)	ns	ns	ns	289 (527)	ns	99 (204)	0.002	67 (124)	ns
Dyslipidemia	488 (754)	ns	ns	ns	337 (498)	ns	106 (189)	ns	68 (114)	0.043
Diabetes	42 (740)	ns	ns	ns	38 (487)	ns	11 (184)	ns	10 (112)	ns
Smokers	251 (743)	ns	ns	ns	174 (492)	0.041	50 (185)	ns	34 (115)	ns
Atrial fibrillation	59 (808)	ns	ns	0.033	46 (527)	ns	23 (204)	<0.001	17 (124)	0.004
Non-calcified and non-stenotic coronary arteries	450 (808)	ns	ns	0.031	269 (527)	ns	123 (204)	0.035	66 (124)	0.045
Over 50% stenosis in coronary arteries	142 (808)	ns	ns	ns	101 (527)	ns	25 (204)	0.037	19 (124)	ns
Prior myocardial infarction	11 (808)	ns	ns	ns	11 (527)	ns	6 (204)	0.009	6 (124)	0.015
Prior stroke or TIA	39 (806)	ns	ns	ns	39 (526)	ns	9 (204)	ns	8 (124)	ns
Moderately severe or severe mitral regurgitation	6 (808)	ns	ns	ns	5 (527)	ns	3 (204)	0.026	2 (124)	0.045
Moderately severe or severe aortic regurgitation	16 (808)	ns	ns	0.010	8 (525)	ns	6 (201)	ns	3 (122)	0.008
LA area, cm^2^	18.8±5.7 (204)	ns	ns	<0.001	19.2±5.6 (124)	ns	NA	NA	NA	NA
Heart failure	12 (808)	ns	ns	0.043	8 (527)	ns	4 (204)	0.010	2 (124)	ns

BMI, Body Mass Index; LA, Left Atrium; LAA, Left Atrial Appendage; NA, not applicable; ns, not significant; TIA, Transient Ischemic Attack.

**Table 3:** Associations between morphological features of the left atrial appendage, the left atrium area, demographic information, and the medical histories of patients without persistent or paroxysmal atrial fibrillation (n = 749).

Characteristic	N, positives or mean±SD (N, total)	LAA morpho-logical class (*P*)	LAA lobes (*P*)	LAA length (*P*)	Relative LAA length		LA area		Relative LA area	
					N, positives or mean±SD (N, total)	*P*	N, positives or mean±SD (N, total)	*P*	N, positives or mean±SD (N, total)	*P*
Females	498 (749)	ns	0.015	<0.001	326 (481)	<0.001	112 (181)	0.002	66 (107)	ns
Overweight (BMI>25)	383 (630)	0.003	ns	ns	NA	NA	79 (147)	<0.001	NA	NA
Obese (BMI>30)	150 (479)	ns	ns	ns	NA	NA	31 (105)	0.001	NA	NA
Body surface area, m^2^	1.92±0.25 (481)	0.018	ns	<0.001	NA	NA	1.95±0.25 (106)	<0.001	NA	NA
Hypertension	374 (749)	ns	ns	ns	260 (481)	ns	84 (181)	0.003	57 (107)	ns
Dyslipidemia	447 (700)	ns	ns	ns	305 (456)	ns	90 (169)	ns	57 (107)	ns
Diabetes	39 (690)	ns	ns	ns	35 (448)	ns	11 (165)	ns	10 (99)	ns
Smokers	231 (688)	ns	ns	ns	158 (449)	0.039	43 (165)	ns	28 (100)	ns
Non-calcified and non-stenotic coronary arteries	423 (749)	ns	ns	0.031	251 (481)	ns	113 (181)	ns	61 (107)	ns
Over 50% stenosis in coronary arteries	128 (749)	ns	ns	ns	89 (481)	ns	20 (181)	ns	14 (107)	ns
Prior myocardial infarction	11 (749)	ns	ns	ns	11 (481)	ns	6 (181)	0.005	6 (107)	0.009
Prior stroke or TIA	31 (747)	ns	ns	ns	22 (480)	ns	8 (181)	ns	7 (107)	ns
Moderately severe or severe mitral regurgitation	5 (749)	ns	ns	ns	4 (481)	ns	3 (181)	0.015	2 (107)	0.034
Moderately severe or severe aortic regurgitation	12 (749)	ns	ns	0.018	4 (479)	ns	3 (178)	ns	0 (105)	NA
LA area	18.1±5.1 (181)	ns	ns	0.001	18.57±5.3 (107)	ns	NA	NA	NA	NA
Heart failure	11 (749)	ns	ns	ns	7 (481)	ns	4 (181)	0.006	2 (107)	ns

BMI, Body Mass Index; LA, Left Atrium; LAA, Left Atrial Appendage; NA, not applicable; ns, not significant; TIA, Transient Ischemic Attack.

**Table 4:** Correlates between features of the left atrial appendage and age classifications.

	N	LAA classes (*P*)	LAA lobes (*P*)	LAA length (*P*)	Relative LAA length		LA area		Relative LA area	
					N	*P*	N	*P*	N	*P*
Age as a continuous variable	808	ns	ns	ns	527	<0.001 ^a^	204	0.034 ^b^	124	<0.001 ^c^
Non-AF patients	749	ns	ns	ns	481	<0.001 ^d^	181	ns	107	<0.001 ^e^
AF patients	59	ns	ns	ns	46	ns	23	ns	17	<0.001 ^f^
Over 53 years ^g^	399	ns	ns	ns	266	0.004	91	ns	124	0.004
Non-AF patients	362	ns	ns	ns	237	0.009	78	ns	107	0.006
AF patients	37	ns	ns	ns	29	ns	13	ns	17	ns
Quartiles according to age ^h^	808	ns	ns	ns	527	0.002	204	ns	124	0.016
Non-AF patients	749	ns	ns	ns	481	0.001	181	ns	107	0.021
AF patients	59	ns	ns	ns	46	ns	23	ns	17	ns
10-year divisions of age ^i^	808	ns	ns	ns	527	0.007	204	ns	124	0.021
Non-AF patients	749	ns	ns	ns	481	0.004	181	ns	107	0.031
AF patients	59	ns	ns	ns	46	ns	23	ns	17	ns

LA, Left Atrium; LAA, Left Atrial Appendage; ns, not significant.

^a^ *r* = 0.156.

^b^ *r* = 0.148.

^c^ *r* = 0.861.

^d^ *r* = 0.162.

^e^ *r* = 0.835.

^f^ *r* = 0.945.

^g^ Age median 53 years; patients over 53 years old compared with younger patients.

^h^ Groups: <48 years (26.3%), 48–53 years (24.3%), 54–58 years (23.8%), ≥59 years (25.6%).

^i^ Groups: <40 years (9.4%), 40–49 years (24.6%), 50–59 years (44.6%), ≥60 years (21.5%).

The proportion of LAAs in each morphological class were: 62.3% (WindSock), 18.6% (Cactus), 10.0% (ChickenWing), and 9.2% (CauliFlower). A greater number of LAA morphological classes with multiple lobes (i.e. Cactus and WindSock) were seen in overweight patients vs. patients of a normal weight (84% vs. 76% (*p* = 0.010)). In addition, gender significantly affected the number of LAA lobes (*p* = 0.007), with men presenting more frequently with multi-lobed LAAs (46% vs. 35%; *p* = 0.003).

Regarding relative LA area, dyslipidemia (10.4±2.9 cm^2^/m^2^ vs. 9.3±2.3 cm^2^/m^2^; p = 0.043), a history of AF (11.7±3.2 cm^2^/m^2^ vs. 9.5±2.3 cm^2^/m^2^; p = 0.004), calcified and/or stenotic coronary arteries (10.4±2.9 cm^2^/m^2^ vs. 9.3±2.3 cm^2^/m^2^; p = 0.045), myocardial infarction (MI, 13.5±4.0 cm^2^/m^2^ vs. 9.6±2.5 cm^2^/m^2^; p = 0.015), and moderately severe/severe mitral (14.0±1.1 cm^2^/m^2^ vs. 9.8±2.6 cm^2^/m^2^; p = 0.045) or aortic regurgitation (15.8±3.1 cm^2^/m^2^ vs. 9.6±2.4 cm^2^/m^2^; p = 0.008), were all associated with an increased relative LA area.

Age, together with the variables shown in Table 2 that correlated with relative LA area were then adjusted in linear regression analyses. With an adjusted R^2^ value of 0.362, age (*p* = 0.003), a history of AF (*p* = 0.021), a history of MI (*p*<0.001), and moderately severe/severe aortic regurgitation (*p*<0.001), all correlated with an increased relative LA area. Similarly, the variables shown in Fig 3 that correlated with relative LAA length were also adjusted in linear regression analyses. Age (*p*<0.001), female gender (*p* = 0.003), and moderately severe/severe aortic/mitral regurgitation (*p* = 0.014), all correlated with an increased relative LAA length, with an adjusted R^2^ value of 0.058.

### Patients without atrial fibrillation

Table 3 shows the associations between LAA morphology and diagnosed medical conditions in patients without AF (n = 749). In terms of relative LAA length, the results were comparable to those obtained for the overall population. In addition, as shown in Table 4, age was a significant correlate for a longer relative length of LAA (*r* = 0.162; *p*<0.001). Regarding the relative LA area, only age (*r* = 0.835; *p*<0.001), a history of MI (13.5±4.0 cm^2^/m^2^ vs. 9.3±2.1 cm^2^/m^2^; *p* = 0.009), and moderately severe/severe mitral regurgitation (14.0±1.1 cm^2^/m^2^ vs. 9.4±2.4 cm^2^/m^2^; *p* = 0.034), remained significant. LA size was found to correlate with LAA length (*r* = 0.251; *p* = 0.001), but not with relative LAA length or any other LAA characteristics.

The proportions of LAA morphological classes were: 61.5% (WindSock), 18.8% (Cactus), 10.0% (ChickenWing), and 9.6% (CauliFlower). Men more frequently exhibited multi-lobed LAAs (45% vs. 35%; *p* = 0.007), with a shorter relative LAA length (2.1±4.8 cm/m^2^ vs. 2.3±0.47 cm/m^2^; *p*<0.001) vs. women. Among those patients with a normal weight, relative LAA length lost its gender-specific association. Similarly, among overweight patients, the multi-lobed status lost its association with gender. The division of morphological classes showed a significant difference (*p* = 0.003) when comparing patients that were overweight versus those of a normal weight, with overweight patients exhibiting a greater number of multi-lobed LAA morphological subtypes (84% vs. 76%; *p* = 0.021).

Excluding those patients with a history of stroke or TIA did not affect any of the previously described data.

## Discussion

We retrospectively assessed LAA morphology in a comprehensive, consecutive study population, deriving correlates for LAA morphology with demographic data, and diagnosed medical conditions. The key findings of our study for the overall population, and non-AF population, were that age and gender influenced relative LAA length, and that men exhibited more multi-lobed LAAs compared to women. Moreover, if the patient was overweight, the multi-lobed LAA form was more prevalent.

In contrast to this study, and its largely non-AF patient population, the majority of previous LAA morphological studies have focused on subjects with AF [18–20]. As far as we are aware, this is the most extensive study to explore LAA morphology in a non-AF population. We analyzed a consecutive series of patients undergoing coronary artery CT angiography, which was undertaken to rule out the presence of coronary artery disease in patients with a low pretest probability of this condition. Our study patients can therefore be considered to be an appropriate representation of the normal population.

CCTA was performed at the mid-diastolic phase and in sinus rhythm. The LAA volume changes dynamically according to the cardiac cycle, with the greatest volume apparent at the ventricular end-systolic phase [21]. Therefore, our results for LAA length likely represent its intermediate value. However, the LAA is known to exhibit more variability in terms of volume and orifice size during the cardiac cycle in patients with sinus rhythm compared to those with AF [22].

### Left atrial appendage morphology

In our study, age, gender, weight, and smoking status all influenced LAA features, with these associations comparable in the overall and non-AF populations.

The frequency of embolic events increases with age [23], although relatively few studies have investigated the possible influence of age on LAA morphology. Aging might alter the properties of the LAA wall as well as other components of the cardiovascular system, with this remodeling increasing the risk of thrombosis. These events present one possible explanation for the higher prevalence of embolic events in elderly patients.

We could find no association between aging and direct morphological features of the LAA. Veinot et al. and Boucebci et al. both reported similar results regarding LAA length and lobe number in smaller non-AF populations (n = 500 and n = 193, respectively) [13,24], with Ilercil et al. examining LAA volume in AF patients [25]. However, after adjusting LAA lengths by BSA values, a positive correlation was identified between LAA length and age. While the study by Boucebci et al. excluded reports on BSA-related LAA length, BSA indexed to LAA volume was found to display an age-related association, agreeing with our data [24].

Men exhibited more multi-lobed and longer LAAs than women, but after adjusting for BSA values, women were found to exhibit significantly longer LAAs. Therefore, the elevated proportion of smokers among our male patients may explain the shorter relative LAA lengths among smokers compared to non-smokers. Boucebci et al. reported that men had longer LAAs, but that lobe number, or BSA-related LAA volume, presented no significant differences between the genders [24]. Veinot et al. also failed to find any gender-specific effects for LAA length, body-size-related LAA length, or the number of LAA lobes [13]. However different amounts of pericardial fat might account for the discordance between studies. After excluding patients with a normal body mass from our analyses, gender specific differences in LAA lobe number were lost. Furthermore, different study methodologies were used. We analyzed LAAs using a multiplanar reconstruction view whereas Boucebci et al. used three-dimensional reconstructions, and Veinot et al. examined autopsied hearts.

The amount of epicardial adipose tissue has been associated with lowered LAA ejection fraction in AF patients which may be attributed to increased numbers of adipocytes and their remodeling effects [26]. We found that overweight patients manifested a greater number of multi-lobed LAA morphological subtypes compared to patients with a normal weight, although there was no association with the actual lobe number. These data suggest that increased pericardial fat in obese subjects leads to a deformation of the LAA wall, thereby remodeling this structure [12].

According to our results, the LAA is significantly longer in patients with severe mitral and/or aortic valve regurgitation vs. patients with no valvular regurgitation. Chronic valvular regurgitation provokes both volume and pressure overload in the left side of the heart [27,28] which may elongate the LAA. Data on mitral or aortic valve stenoses were, unfortunately, unavailable for our patients. The number of patients with significant valve stenosis was very low since mitral stenosis is extremely rare in the Finnish Caucasian population, and CCTA is not included in the preoperative evaluation of patients with aortic stenosis.

The AF patients had markedly elongated LAAs, but this difference (between AF and non-AF patients) disappeared after indexing to BSA values. Relatively few studies have investigated LAA length in patients with AF but without a history of stroke. AF patients were found to display larger LAA volumes and greater LAA orifice dimensions, although only in smaller, non-BSA-indexed patient populations (n = 34 and n = 46) [6,29]. In a short-term (2.5 months) follow-up study regarding recent onset AF, non-BSA-indexed LAA length exhibited no significant increase between the measurement dates [30].

### Left atrium size

We found that men had a larger LA area than women, and that BSA positively correlated with LA area. These results indicate that increased body mass imposes more pressure on the LA, as reported in an earlier study on male gender and obesity [31,32]. Predictably, the difference between genders disappeared after values for LA area were BSA-indexed, paralleling the results of Maceira et al. [33]. Thereafter, we found that aging, prior MI, and aortic/mitral regurgitation were associated with increased relative LA area in both study groups. Furthermore, AF patients also possessed larger relative LA areas.

Age positively correlated with increased relative LA area in the overall study population, in non-AF patients, and in AF patients. These data contradict several earlier studies. Aurigemma et al. found no association between BSA indexed LA volume and healthy aging [34]. In contrast to our study, none of their study patients (n = 230) had been diagnosed with cardiovascular diseases. Other studies have reported that aging correlates with decreased relative LA diameter in healthy patients [33], and similar LA volumes (not BSA indexed) in AF patients [25]. Moreover, larger LA areas are suggested to be a result of an underlying pathological dysfunction rather than healthy aging [35,36].

Several etiological factors can cause enlargement of the LA, i.e. AF, mitral regurgitation, hypertension, and other causes of heart failure [29,36–39]. LA enlargement typically leads to increased pressure on the LAA. In our study, an increased LA size was associated with elongation in both AF and non-AF patients. However, after LAA length indexing to BSA, this correlation disappeared.

The relative LA area was significantly larger in patients with severe aortic or mitral valve regurgitation compared to patients with no regurgitation, as suggested previously [27,28]. In addition, a history of MI has been indicated to cause long-term dilation of the left atrium [40], which was also recognized in the present study.

Based on our results, we could hypothesize that remodeling of the LAA may be involved in cardioembolic events. First, as the lumen of the LAA is enlarged and reshaped due to fibrotic degeneration or other causes, this process may alter the hemodynamic profile inside LAA predisposing to clot formation even without AF. Second, as these structure walls stretch, electrical conduction of the heart is deteriorated resulting eventually in arrhythmia. The AF may thus follow atrial and LAA enlargement [41,42]. Earlier studies have noted that enlarged LAs [41] and LAAs [43] are prevalent also in non-AF stroke patients. AF might therefore represent one essential but not an exclusive factor in the etiology of cardioembolic events.

### Limitations

Our study has limitations. Women were overrepresented in our study population, and our subgroup of AF patients was small compared to the non-AF subgroup, which may have biased comparisons. All patients were imaged during mid-diastole, which is not an optimal phase with which to observe maximal LAA length, although it does optimize morphological imaging. CCTA imaging during the end-systolic phase would have resulted in non-diagnostic coronary images and could therefore not be justified.

While only one observer assessed LAA morphological features, intra-observer and inter-observer reproducibility proved to be good in an earlier study [44]. The retrospective assessment of weight status was challenging. In some cases this classification was based on BMI, which was not necessarily registered at the time of CCTA evaluation. Furthermore, it was difficult to make an accurate assessment of the degree of stenosis in calcified coronary arteries using CCTA.

## Conclusions

Our study findings among adults suggest the following: aging demonstrates a positive correlation with BSA-related LAA length, the female gender is associated with increased relative LAA length, and that multi-lobed LAAs are more frequent in male patients. Overweight patients may possess more LAA morphological classes with multiple lobes. The relative LAA length and number of LAA lobes seems to be unaffected by a history of AF, or increased LA size. Cardioembolic events may not be exclusively tied to fibrillation, and progressive enlargement and fibrotic degeneration of the LAA may be of clinical importance.

## References

[1] Telman G, Kouperberg E, Sprecher E, Yarnitsky D. Distribution of etiologies in patients above and below age 45 with first-ever ischemic stroke. Acta Neurol Scand. 2008;117: 311–316. 10.1111/j.1600-0404.2007.00953.x 18042269

[2] Hart RG, Diener HC, Coutts SB, Easton JD, Granger CB, O'Donnell MJ, et al Embolic strokes of undetermined source: the case for a new clinical construct. Lancet Neurol. 2014;13: 429–438. 10.1016/S1474-4422(13)70310-7 24646875

[3] Holmes DR, Reddy VY, Turi ZG, Doshi SK, Sievert H, Buchbinder M, et al Percutaneous closure of the left atrial appendage versus warfarin therapy for prevention of stroke in patients with atrial fibrillation: a randomised non-inferiority trial. Lancet. 2009;374: 534–542. 10.1016/S0140-6736(09)61343-X 19683639

[4] Romero J, Cao JJ, Garcia MJ, Taub CC. Cardiac imaging for assessment of left atrial appendage stasis and thrombosis. Nat Rev Cardiol. 2014;11: 470–80. 10.1038/nrcardio.2014.77 24913058

[5] Wolf PA, Abbott RD, Kannel WB. Atrial fibrillation as an independent risk factor for stroke: The Framingham Study. Stroke 1991;22: 983–988. 1866765 10.1161/01.str.22.8.983

[6] Imada M, Funabashi N, Asano M, Uehara M, Ueda M, Komuro I. Anatomical remodeling of left atria in subjects with chronic and paroxysmal atrial fibrillation evaluated by multislice computed tomography. Int J Cardiol. 2007;119: 384–388. 10.1016/j.ijcard.2006.07.162 17064785

[7] Yamamoto M, Seo Y, Kawamatsu N, Sato K, Sugano A, Machino-Ohtsuka T, et al Complex left atrial appendage morphology and left atrial appendage thrombus formation in patients with atrial fibrillation. Circ Cardiovasc Imaging. 2014;7: 337–343. 10.1161/CIRCIMAGING.113.001317 24523417

[8] Kimura T, Takatsuki S, Inagawa K, Katsumata Y, Nishiyama T, Nishiyama N, et al Anatomical characteristics of the left atrial appendage in cardiogenic stroke with low CHADS2 scores. Heart Rhythm. 2013;10: 921–925. 10.1016/j.hrthm.2013.01.036 23384894

[9] Di Biase L, Santangeli P, Anselmino M, Mohanty P, Salvetti I, Gili S, et al Does the left atrial appendage morphology correlate with the risk of stroke in patients with atrial fibrillation? Results from a multicenter study. J Am Coll Cardiol. 2012;60: 531–538. 10.1016/j.jacc.2012.04.032 22858289

[10] Cohoon KP, McBane RD, Ammash N, Slusser JP, Grill DE, Wysokinski WE. Relationship between body mass index and left atrial appendage thrombus in nonvalvular atrial fibrillation. J Thromb Thrombolysis. 2015;141: 1–6. 10.1007/s11239-015-1266-7 26282111

[11] Tabata T, Oki T, Fukuda N, Iuchi A, Manabe K, Kageji Y, et al Influence of left atrial pressure on left atrial appendage flow velocity patterns in patients in sinus rhythm. J Am Soc Echocardiogr. 1996;9: 857–864. 8943446 10.1016/s0894-7317(96)90478-2

[12] Pan NH, Tsao HM, Chang NC, Chen YJ, Chen SA. Aging dilates atrium and pulmonary veins: implications for the genesis of atrial fibrillation. Chest. 2008;133: 190–196. 10.1378/chest.07-1769 18187745

[13] Veinot JP, Harrity PJ, Gentile F, Khandheria BK, Bailey KR, Eickholt JT, et al Anatomy of the normal left atrial appendage: a quantitative study of age-related changes in 500 autopsy hearts: implications for echocardiographic examination. Circulation. 1997;96: 3112–3115. 9386182 10.1161/01.cir.96.9.3112

[14] Iozzo P. Myocardial, perivascular, and epicardial fat. Diabetes Care. 2011;34 Suppl 2:s371–379. 21525485 10.2337/dc11-s250 PMC3632210

[15] Sabarudin A, Sun Z. Radiation dose measurements in coronary CT angiography. World J Cardiol. 2013;5: 459–464. 10.4330/wjc.v5.i12.459 24392190 PMC3879695

[16] Wang Y, Di Biase L, Horton RP, Nguyen T, Morhanty P, Natale A. Left atrial appendage studied by computed tomography to help plan for appendage closure device placement. J Cardiovasc Electrophysiol. 2010;21: 973–982. 10.1111/j.1540-8167.2010.01814.x 20550614

[17] Mosteller RD. Simplified calculation of body surface area. N Engl J Med. 1987;317: 1098 10.1056/NEJM198710223171717 3657876

[18] Nedios S, Kornej J, Koutalas E, Bertagnolli L, Kosiuk J, Rolf S, et al Left atrial appendage morphology and thromboembolic risk after catheter ablation for atrial fibrillation. Heart Rhythm. 2014;11: 2239–2246 10.1016/j.hrthm.2014.08.016 25128733

[19] Khurram IM, Dewire J, Mager M, Maqbool F, Zimmerman SL, Zipunnikov V, et al Relationship between left atrial appendage morphology and stroke in patients with atrial fibrillation. Heart Rhythm. 2013;10: 1843–1849. 10.1016/j.hrthm.2013.09.065 24076444

[20] Fukushima K, Fukushima N, Kato K, Ejima K, Sato H, Fukushima K, et al Correlation between left atrial appendage morphology and flow velocity in patients with paroxysmal atrial fibrillation. Eur Heart J Cardiovasc Imaging. 2016;17: 59–66. 10.1093/ehjci/jev117 25944049

[21] Li CY, Gao BL, Liu XW, Fan QY, Zhang XJ, Liu GC, et al Quantitative Evaluation of the Substantially Variable Morphology and Function of the Left Atrial Appendage and Its Relation with Adjacent Structures. PLoS ONE. 2015;10: e0126818 10.1371/journal.pone.0126818 26230395 PMC4521946

[22] Beigel R, Wunderlich NC, Ho SY, Arsanjani R, Siegel RJ. The left atrial appendage: anatomy, function, and noninvasive evaluation. JACC Cardiovasc Imaging. 2014;7: 1251–1265. 10.1016/j.jcmg.2014.08.009 25496544

[23] Mozaffarian D, Benjamin EJ, Go AS, Arnett DK, Blaha MJ, Cushman M, et al Heart disease and stroke statistics—2016 update: a report from the American Heart Association. Circulation. 2016;133: e38–e360. 10.1161/CIR.0000000000000350 26673558

[24] Boucebci S, Pambrun T, Velasco S, Duboe PO, Ingrand P, Tasu JP. Assessment of normal left atrial appendage anatomy and function over gender and ages by dynamic cardiac CT. Eur Radiol. 2016;26: 1512–1520. 10.1007/s00330-015-3962-2 26310584

[25] Ilercil A, Kondapaneni J, Hla A, Shirani J. Influence of age on left atrial appendage function in patients with nonvalvular atrial fibrillation. Clin Cardiol. 2001;24: 39–44. 11195605 10.1002/clc.4960240107 PMC6654982

[26] Tsao HM, Hu WC, Tsai PH, Lee CL, Liu FC, Wang HH, et al The Abundance of Epicardial Adipose Tissue Surrounding Left Atrium Is Associated With the Occurrence of Stroke in Patients With Atrial Fibrillation. Medicine (Baltimore). 2016;95: e3260. 27057876 10.1097/MD.0000000000003260 PMC4998792

[27] Gehl LG, Mintz GS, Kotler MN, Segal BL. Left atrial volume overload in mitral regurgitation: A two dimensional echocardiographic study. Am J Cardiol. 1982;49: 33–38. 7053608 10.1016/0002-9149(82)90274-0

[28] Gotzmann M, Lindstaedt M, Mügge A. From pressure overload to volume overload: aortic regurgitation after transcatheter aortic valve implantation. Am Heart J. 2012;163: 903–911. 10.1016/j.ahj.2012.03.017 22709742

[29] Shirani J, Alaeddini J. Structural remodeling of the left atrial appendage in patients with chronic non-valvular atrial fibrillation: Implications for thrombus formation, systemic embolism, and assessment by transesophageal echocardiography. Cardiovasc Pathol. 2000;9: 95–101. 10867359 10.1016/s1054-8807(00)00030-2

[30] Weigner MJ, Katz SE, Douglas PS, Manning WJ. Left atrial appendage anatomy and function: short term response to sustained atrial fibrillation. Heart. 1999;82: 555–558. 10525507 10.1136/hrt.82.5.555 PMC1760787

[31] Ploumen MA, Baur LH, Streppel MJ, Lodewijks-van der Bolt CL, Winkens B, Winkens RA, Stoffers HE. Age is an independent risk factor for left atrial dysfunction: results from an observational study. Neth Heart J. 2010;18: 243–247. 20505797 10.1007/BF03091770 PMC2871744

[32] Movahed MR, Saito Y. Obesity is associated with left atrial enlargement, E/A reversal and left ventricular hypertrophy. Exp Clin Cardiol. 2008;13: 89–91. 19343123 PMC2586403

[33] Maceira AM, Cosín-Sales J, Roughton M, Prasad SK, Pennell DJ. Reference left atrial dimensions and volumes by steady state free precession cardiovascular magnetic resonance. J Cardiovasc Magn Reson. 2010;12: 65 10.1186/1532-429X-12-65 21070636 PMC2994941

[34] Aurigemma GP, Gottdiener JS, Arnold AM, Chinali M, Hill JC, Kitzman D. Left atrial volume and geometry in healthy aging: the Cardiovascular Health Study. Circ Cardiovasc Imaging. 2009;2: 282–289. 10.1161/CIRCIMAGING.108.826602 19808608 PMC4156514

[35] Faustino A, Providência R, Barra S, Paiva L, Trigo J, Botelho A, et al Which method of left atrium size quantification is the most accurate to recognize thromboembolic risk in patients with non-valvular atrial fibrillation? Cardiovasc Ultrasound. 2014;12: 28 10.1186/1476-7120-12-28 25052699 PMC4121510

[36] Casaclang-Verzosa G, Gersh BJ, Tsang TS. Structural and functional remodeling of the left atrium: clinical and therapeutic implications for atrial fibrillation. J Am Coll Cardiol. 2008;51: 1–11. 10.1016/j.jacc.2007.09.026 18174029

[37] Sanfilippo AJ, Abascal VM, Sheehan M, Oertel LB, Harrigan P, Hughes RA, et al Atrial enlargement as a consequence of atrial fibrillation. A prospective echocardiographic study. Circulation. 1990;82: 792–797. 2144217 10.1161/01.cir.82.3.792

[38] Ma X, Zhang X, Guo W. Factors to Predict Recurrence of Atrial Fibrillation in Patients with Hypertension. Clin Cardiol. 2009;32: 264–268. 10.1002/clc.20449 19452484 PMC6653222

[39] Kennedy JW, Yarnall SR, Murray JA, Figley MM. Quantitative angiography, IV: relationships of left atrial and ventricular pressure and volume in mitral valve disease. Circulation. 1970;41: 817–824. 5429491 10.1161/01.cir.41.5.817

[40] Popescu BA, Macor F, Antonini-Canterin F, Giannuzzi P, Temporelli PL, Bosimini E, et al Left atrium remodeling after acute myocardial infarction (results of the GISSI-3 Echo Substudy). Am J Cardiol. 2004;93: 1156–1159. 10.1016/j.amjcard.2004.01.046 15110211

[41] Patel DA, Lavie CJ, Milani RV, Shah S, Gilliland Y. Clinical Implications of Left Atrial Enlargement: A Review. Ochsner J. 2009;9: 191–196. 21603443 PMC3096293

[42] Di Biase L, Burkhardt JD, Mohanty P, Sanchez J, Mohanty S, Horton R, et al Left atrial appendage: an underrecognized trigger site of atrial fibrillation. Circulation. 2010;122: 109–118. 10.1161/CIRCULATIONAHA.109.928903 20606120

[43] Taina M, Vanninen R, Hedman M, Jäkälä P, Kärkkäinen S, Tapiola T, et al Left Atrial Appendage Volume Increased in More Than Half of Patients with Cryptogenic Stroke. PLoS ONE. 2013;8: e79519 10.1371/journal.pone.0079519 24223960 PMC3817123

[44] Korhonen M, Muuronen A, Arponen O, Mustonen P, Hedman M, Jäkälä P, et al Left atrial appendage morphology in patients with suspected cardiogenic stroke without known atrial fibrillation. PLoS ONE. 2015;10: e0118822 10.1371/journal.pone.0118822 25751618 PMC4353716

